# Reply to RE: Should LHRH therapy be continued in patients receiving Abiraterone Acetate?

**DOI:** 10.1038/s41391-022-00617-3

**Published:** 2022-11-29

**Authors:** Carsten-Henning Ohlmann

**Affiliations:** 1 Johanniter Kliniken Bonn, Johanniterstr. 3-5, 53113 Bonn, Germany; 2 Department of Urology and Pediatric Urology, Saarland University, Kirrberger Street 100, 66421 Homburg/Saar, Germany

**Keywords:** Cancer therapy, Prostate cancer

## To the Editor:

Ah-Thiane and Supiot [1] commented on our recent publication about the SPARE-trial [2] and we are grateful for the fruitful comments.

The CARLHA trial, published by Supiot et al. [3], was undoubtedly the first trial to evaluate the effect of abiraterone (AA) alone in patients with biochemically-relapsing prostate cancer patients. Comparable to the LACOG-0415 trial, the CARLHA trial was conducted in the setting of castration-naive patients revealing that treatment with AA alone does not achieve castrate levels of testosterone in every patient. However, both trials recruited patients in a disease state that should be clearly distinguished from the SPARE-trial, which was undertaken in castration-resistant prostate cancer (CRPC) patients. Any result from the CARLHA, LACOG-0415 and the SPARE trial should be interpreted with caution in view of this important difference. This difference may account for the seemingly contradicting results regarding serum-testosterone levels measured under treatment in the trials. These results should not be regarded as conflicting but should prompt us to further evaluate the different disease states with regard to the efficacy of AA on steroidogenesis and mechanisms of resistance.

The elevated serum levels of LH and FSH measured in the SPARE-trial, similar to the results in the CARLHA trial, may impair the efficacy of AA and display an unpredictable hazard to patients receiving AA without ADT - leading to early progression, and consequently shortened cancer-specific and overall survival as outlined in the SPARE-protocol [4].

However, LH acts mainly on prostate cancer cells by causing an increase in the expression of several key steroidogenic enzymes including CYP11A1, CYP17A1 and CYP19A1 [5]. Silencing the LH receptor suppresses androgen synthesis [6]. We therefore conclude that the constant low levels of serum testosterone < 0.029 ng/ml in the SPARE trial demonstrate that the steroidogenic potential of LH may not be clinically relevant in CRPC patients treated with AA alone. Even though FSH may drive transition of prostate-cancer cells to castration-resistance in an androgen-independent manner, its role in CRPC cells is a matter of debate. In view of the exploratory results of the SPARE-trial showing no difference in radiographic-progression free survival at 12 months the effect of elevated serum LH and FSH levels noticed may also be of minor clinical importance.

With regard to efficacy and toxicity, the exploratory SPARE trial was not powered to show any difference in any of the endpoints analyzed. Quality of life was not evaluated due to lack of power, whereas analysis of toxicity was mandatory by health care authorities but failed to show any difference.

In summary, as clearly stated in the conclusions, the exploratory results of the SPARE trial warrant further confirmatory trials to demonstrate the efficacy of AA treatment without continuous LHRH-therapy in mCRPC patients. The results of the CARLHA and the LACOG-0415 trial display comparable drawbacks and limitations being phase-I and II trials lacking statistical power. However, all three trials were driven by the same vision of identifying ADT-free treatment options for prostate cancer patients, which is a desirable aim due to toxicity and costs of ADT.
